# Descriptive Study on Epidemiology, Clinical Presentation, Treatment, and Outcome of Supracondylar Fractures Treated in a Base Hospital of Sri Lanka: A Single-Center Study

**DOI:** 10.7759/cureus.40494

**Published:** 2023-06-16

**Authors:** Fathima S Mubarak, Muhammed A Mohamed Anzar, Kandeepan Kanagaratnam

**Affiliations:** 1 Cardiothoracic Surgery, Harefield Hospital, Harefield, GBR; 2 Orthopedic Surgery, Ashraff Memorial Hospital, Kalmunai, LKA

**Keywords:** median nerve injury, hospital based, ortho surgery, surgically managed, free surgery, native medicine, sri lanka, pediatric orthopedic surgery, pediatric fractures, distal humerus fractures

## Abstract

Introduction: Supracondylar fractures are common pediatric elbow injuries, with management in developing countries presenting challenges due to limited resources, inadequate facilities, and a lack of trained personnel.

Method: This study aimed to describe the incidence, demographics, clinical presentation, treatment methods, and outcomes of supracondylar fractures treated at Ashraff Memorial Hospital (AMH)-Kalmunai, a base hospital in Sri Lanka. This is a retrospective descriptive study conducted between January 2019 and December 2020.

Results: The study involved 79 children with supracondylar fractures. The majority of the children were male (70.9%) and their ages ranged from one to 15 years. The study identified falls as the most common presenting complaint (92.4%), followed by road traffic accidents (3.8%), native treatment (2.5%), and mismanagement (1.3%). The majority of fractures affected the right side (69.6%), while the remaining cases involved the left side (30.4%). Regarding the time duration from fracture to hospital presentation, a significant proportion of children sought medical attention on the same day (51.9%), followed by presentations within the first three days (38%), within a week (5.1%), or after a month (3.8%). Based on the Gartland classification, type I fractures accounted for 44.3% of cases, followed by type II fractures (29.1%) and type III fractures (26.6%). The most common treatment approach was closed reduction and percutaneous pinning (41.8%). Other treatment options included plaster of Paris (POP) cast without manipulation (36.7%), POP cast with manipulation (7.6%), analgesics alone (6.3%), and open reduction fixation (5.1%). Follow-up procedures varied, with routine cast removal (11.4%), routine cast and K-wire removal (45.6%), and re-do surgery with routine follow-up (1.3%). Among the type III fractures, two children presented with vascular compromise and anterior interosseous nerve (AIN) impairment, while another two children had AIN impairment only. Type I and type II fractures did not exhibit nerve involvement or vascular impairment. Only one out of the 79 children had an open fracture.

Conclusion: Supracondylar fracture is the most common orthopedic fracture in children. The study sheds light on the challenges and opportunities associated with treating pediatric supracondylar fractures in a resource-constrained context. The findings can help produce guidelines for the management of supracondylar fractures in underdeveloped nations, as well as contribute to global efforts to enhance the management of pediatric fractures.

## Introduction

Supracondylar fractures are one of the most common pediatric elbow injuries worldwide, accounting for approximately 60% of all elbow fractures in children [1]. Supracondylar fractures can result from various mechanisms, such as falls on an outstretched hand or a direct blow to the elbow [2]. These fractures are characterized by a fracture line above the condyles of the humerus, typically occurring in the distal humerus region. They are often classified according to the Gartland classification system, which categorizes fractures as non-displaced (type I), partially displaced (type II), or completely displaced (type III) [3].

The management of supracondylar fractures is well established in developed countries, with most patients receiving timely and appropriate treatment leading to good outcomes [4]. However, in developing countries, the management of supracondylar fractures can be challenging due to factors such as limited resources, inadequate facilities, and a lack of trained personnel [5]. As a result, the treatment and outcome of supracondylar fractures in developing countries may differ from those in developed countries [6]. Therefore, understanding the management of supracondylar fractures in a developing country is important for improving the quality of care for this patient population.

Base hospitals play a vital role in providing healthcare services in developing countries. However, little is known about the epidemiology, clinical presentation, treatment, and outcome of supracondylar fractures treated at base hospitals in developing countries [7]. This study aims to describe the incidence, demographics, clinical presentation, treatment methods, and outcomes of supracondylar fractures treated at a base hospital in a developing country. By describing the management of supracondylar fractures at a base hospital, this study will provide insights into the challenges and opportunities of providing care for these patients in a resource-limited setting. The results of this study will inform the development of guidelines for the management of supracondylar fractures in developing countries and guide policy-makers and healthcare professionals in improving the quality of care for these patients.

## Materials and methods

The study was a retrospective descriptive study conducted at Ashraff Memorial Hospital (AMH)-Kalmunai, a base hospital in Sri Lanka.

Study design

This descriptive study utilized a retrospective design to gather data from the medical records of patients who presented with supracondylar fractures at the base hospital. A retrospective approach was chosen to capture a sufficient sample size and evaluate the past cases treated in the hospital.

Participant selection

The study covered all pediatric patients (ages 1-15) who presented with supracondylar fractures between January 1st, 2019, and December 31st, 2020. 

Data collection

Data were collected through the hospital records, including patient demographics, fracture characteristics, mechanism of injury, clinical presentation, treatment modalities employed, complications, and follow-up outcomes. Data were gathered from the AMH Orthopedic Unit electronic database after the ethical clearance, which is a patient management system used at the hospital. 

Variables measured

The study aimed to collect information on various variables related to supracondylar fractures, including age, gender, date of presentation, side of the fracture, Gartland classification, mechanism of injury, associated injuries, time to presentation, clinical signs and symptoms, radiographic findings, treatment methods (closed reduction, open reduction, immobilization), duration of hospital stay, and short-term outcomes (such as range of motion, complications, and need for further interventions).

Data analysis

The information gathered was entered into a pre-designed proforma built exclusively for the study. The research team examined and validated the proforma, and the data were double-checked for accuracy and completeness. The data were analyzed using statistical software, and the results were provided in the form of descriptive statistics such as frequencies, percentages, means, and standard deviations. 

Ethical considerations

The study adhered to ethical guidelines and obtained approval from the appropriate ethics committee or institutional review board. Patient confidentiality and privacy were ensured throughout the data collection process. Personal identifiers were removed or anonymized to maintain data confidentiality.

## Results

**Figure 1:**
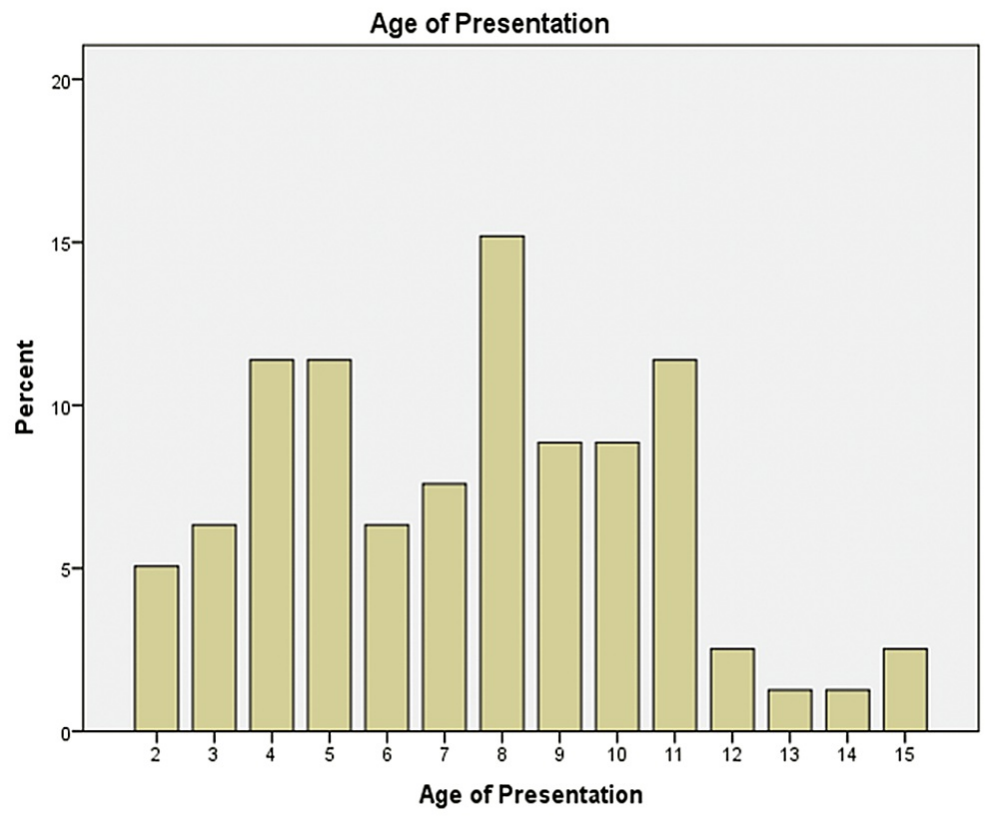
Age of Presentation Age at presentation to the hospital has been depicted in a bar chart.

**Figure 2:**
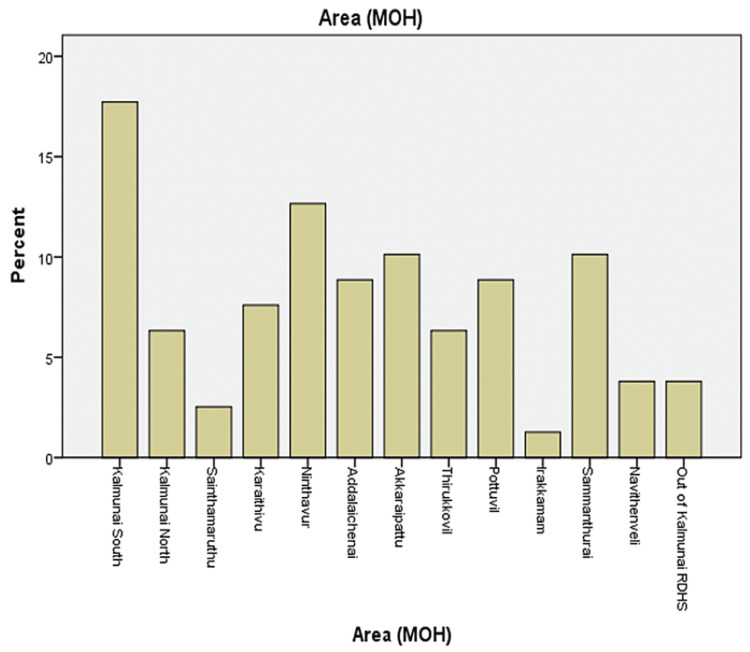
Ministry of Health (MOH) area of the patients The Kalmunai Regional Director of Health Service oversees 13 MOH areas. This bar chart depicts the geographic location of the patients. Kalmunai South, Kalmunai North, Sainthamaruthu, Karaithivu, Nintavur, Addaalachenai, Akkaraipattu, Thirukovil, Pottuvil, Irakkamam, Sammanthurai, and Navaneethavanai are the areas.

The sample consisted of 79 children, 70.9% (n=56) male and 29.1% (n=23) female varying from age 1 to 15 (Figure 1). Epidemiological distribution was categorized according to 13 Kalmunai Regional Director of Health Services (RDHS) divisions and outside of Kalmunai RDHS. 96.2% presented from the Kalmunai RDHS region and the rest (3.8%) were from out of Kalmunai RDHS. While categorizing presentation from Kalmunai RDHS, 17.7 % (n=14) were from Kalmunai South, 6.3% (n=5) from Kalmunai North, 2.5% (n=2) from Sainthamaruthu, 7.6% (n=6) from Karaithivu, 12.7% (n=10) from Nintavur, 8.9% (n=7) from Addaalachenai, 10.1 (n=8) from Akkaraipattu, 6.3% (n=5) from Thirukovil, 8.9% (n=7) from Pottuvil, 1.3% (n=1) from Irakkamam, 10.1% (n=8) from Sammanthurai, and 3.8% (n=3) (Figure 2).

**Figure 3:**
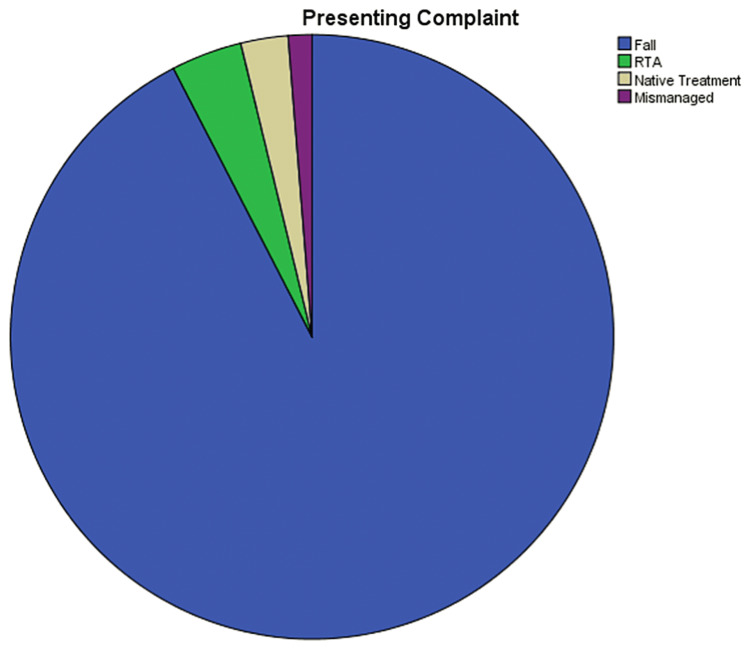
Presenting complaint of the patient This pie chart illustrates the percentage of patients who arrive with supracondylar fractures. They were classified as falls, automobile accidents, native treatment, and mismanagement. RTA, road traffic accident.

**Figure 4:**
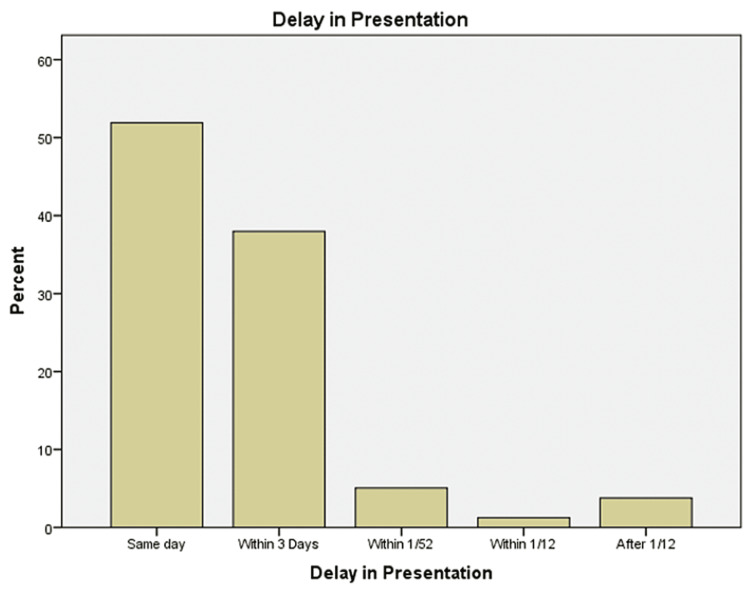
Delay in Presentation Time duration from the fracture to the presentation to the hospital has been depicted here.

Fall was the main presenting complaint at 92.4% (n=73), followed by road traffic accident (RTA) at 3.8% (n=3), following native treatment at 2.5% (n=2), and following mismanagement at 1.3% (n=1) (Figure 3). The majority of the impact was on the right side 69.6% (n=55) while the rest of them were on the left 30.4% (n=24). 51.9 % (41) of the kids presented on the same day; however, 38% (n=30) presented within the first three days, 5.1% (n=4) presented within a week, and 3.8% (n=3) showed a significant delay in the presentation by presenting after a week's duration since the impact (Figure 4).

**Figure 5:**
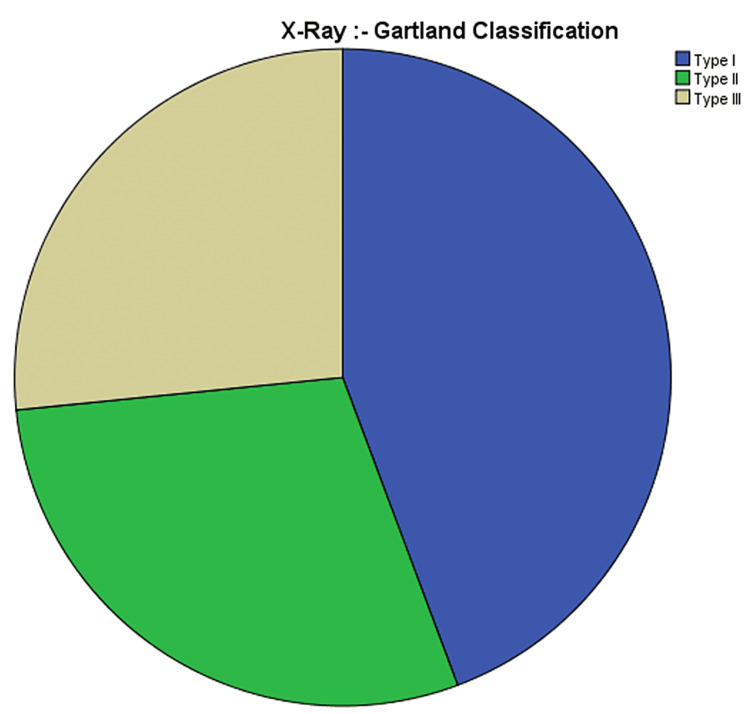
Presentation of the patients according to the Gartland Classification

**Figure 6:**
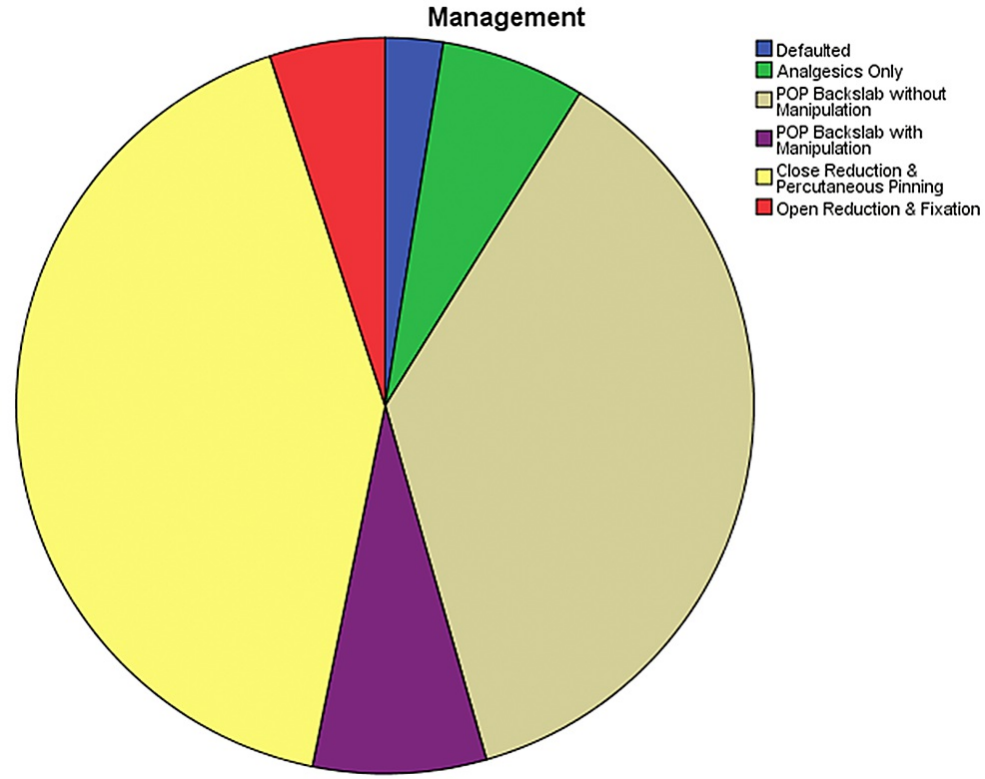
Management and treatment provided for the supracondylar fractures This pie chart represents the percentage of patient management completed. Default therapy, analgesia only, cast without manipulation, cast with manipulation, closed reduction and percutaneous pinning, and open reduction and fixing are among the categories. POP, Plaster of Paris.

**Figure 7:**
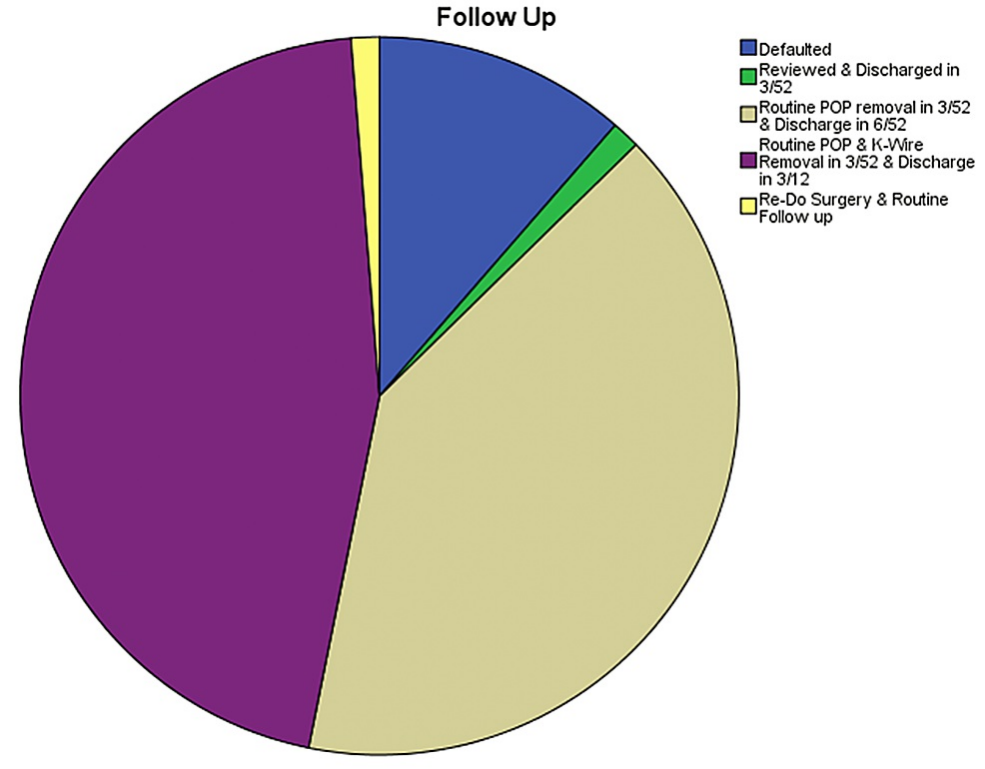
Follow-up percentages of the supracondylar fractures POP, Plaster of Paris.

Gartland classification was used to classify the type of fractures. 44.3% (n=35) of them belonged to type I, 29.1% (n=23) were type II, and 26.6% (n=21) were type III (Figure 5). 2.5% (n=2) left against medical advice (defaulted), 6.3% (n=5) were managed with analgesics only, 36.7% (n=29) were managed with Plaster of Paris (POP) back slab without manipulation,7.6% (n=6) were managed with POP back slab with manipulation, 41.8% (n=33) were managed with closed reduction and percutaneous pinning, and 5.1% (n=4) were managed with open reduction fixation (Figure 6). Regarding follow-up, 11.4% (n=9) defaulted follow-up, 1.3% (n=1) were reviewed and discharged in three weeks, 40.5% (n=32) were routine POP removal in three weeks and discharged in six weeks, 45.6% (n=36) were routine POP and K wire removal in three weeks and discharged in three months, and 1.3% (n=1) underwent re-do surgery and routine follow-up (Figure 7).

There were no nerve involvement or vascular impairment noted in Garland type I and II fractures. However, out of 21 children with type III fractures, two of them presented with vascular compromise and anterior interosseous nerve (AIN) impairment, and another two presented with only AIN impairment. Out of all the 79 children with supracondylar fractures only one presented with an open fracture (Table 1).

**Table 1:** Neurovascular status on presentation AIN, anterior intraosseous nerve.

Gartland classification	Anterior intraosseous nerve involvement only	Pulseless limb only	Anterior intraosseous nerve involvement with pulseless limb	Open fracture
Type I (n=35)	Nil	Nil	Nil	Nil
Type II (n=23)	Nil	Nil	Nil	Nil
Type III (n=21)	2	Nil	2	1

## Discussion

The AMH-Kalmunai provides medical services not only in its region but also to patients from outside the region. In 2019-2020, 3.8% of the total supracondylar fracture patients treated at AMH were from outside the RDHS Kalmunai region.

Supracondylar fractures tend to occur more frequently in males than females, with a male-to-female ratio of 2.4:1. In terms of the side of the elbow affected, two-thirds of patients presented with right-side fractures, while only one-third presented with left-side fractures [8-10].

Most of the patients with supracondylar fractures (92.4%) presented following falls at home, school, or playground. RTAs were the second most common cause [11]. Interestingly, 2.5% of the patients had sought native treatment before presenting to AMH, which is a significant finding in the eastern region of the country. Half of the patients presented with supracondylar fractures within the same day of injury, while 3.8% presented after a month of injury. Late presentation of patients could be due to various reasons such as getting native treatment or being mismanaged at a different center prior to admission to AMH.

The Gartland classification is a commonly used classification system for supracondylar fractures. Type I and type II fractures typically have intact neurovascular status, while type III fractures can result in AIN neuropraxia and the absence of a distal pulse [12]. Most type III fractures needed closed reduction and percutaneous pinning [13]. The follow-up of the patients in the region was satisfactory, and neurological improvement was observed in the patients who presented with neurovascular compromise [14].

The study is significant as it sheds light on the management of supracondylar fractures in a resource-limited setting. The findings of this study will provide important information to healthcare professionals and policymakers not only in Sri Lanka but also in other developing countries facing similar challenges in providing care for pediatric fractures [15].

However, there are certain limitations to the study. First, the study was conducted in a single hospital, which may limit the generalizability of the findings to other hospitals in Sri Lanka or other developing countries. Second, the study was retrospective, which may limit the accuracy and completeness of the data collected. Third, the study only covered the period between January 1st, 2019, and December 31st, 2020, which may not be representative of the management of supracondylar fractures in the hospital or the country as a whole. Fourth, the study did not compare the management of supracondylar fractures at the base hospital with that at other hospitals, which may limit the ability to identify specific challenges and opportunities in the management of supracondylar fractures at the base hospital.

## Conclusions

A supracondylar fracture is a common elbow injury that accounts for approximately 60% of all pediatric elbow fractures worldwide. Although supracondylar fracture management is well established in industrialized countries, it may be difficult in less developed countries due to a lack of funds, inadequate facilities, and a lack of experienced medical practitioners. Understanding the epidemiology, clinical presentation, therapy, and prognosis of supracondylar fractures at these hospitals is critical for improving the level of care offered to this patient population. 

The studies' findings showed that there is a significant frequency of the condition in the region, but there are no suitable protocols or management standards in place. Future studies should focus on collaboration with research institutions and experts to inform protocol development and conduct pilot testing for refinement.
